# Mechanisms of childhood trauma: an integrative review of a multimodal, transdiagnostic pathway

**DOI:** 10.1016/j.ynstr.2025.100737

**Published:** 2025-06-03

**Authors:** J.M. Pasteuning, C. Broeder, T.A.A. Broeders, R.G.G. Busby, A.W. Gathier, E. Kuzminskaite, F. Linsen, C.P. Souama, J.E. Verhoeven, M.S.C. Sep, C.H. Vinkers

**Affiliations:** a Amsterdam UMC location Vrije Universiteit Amsterdam, Department of Psychiatry, Boelelaan, 1117, Amsterdam, the Netherlands; b Amsterdam UMC location Vrije Universiteit Amsterdam, Department of Anatomy & Neurosciences, Boelelaan, 1117, Amsterdam, the Netherlands; c GGZ InGeest Mental Health Care, Amsterdam, the Netherlands; d Amsterdam Neuroscience, Mood, Anxiety, Psychosis, Sleep & Stress Program, Amsterdam, the Netherlands; e Amsterdam Public Health, Mental Health Program, Amsterdam, the Netherlands; f Department of Clinical Psychology, Institute of Psychology, Leiden University, Leiden, the Netherlands

**Keywords:** Childhood trauma, Mental health, Somatic health, Transdiagnostic, Multimodal, Mechanisms

## Abstract

Childhood trauma (CT), conceptualized as emotional, physical or sexual abuse or emotional or physical neglect before the age of 18, is a risk factor for the emergence and poorer course of many mental and somatic disorders. The mechanisms underlying the impact of CT range from (neuro)biological changes (e.g., epigenetics, hypothalamic–pituitary–adrenal axis, and brain structure/function) to psychosocial mechanisms (e.g., personality, attachment, emotion regulation, and coping), and behavioral factors (e.g., smoking and exercise). Given the interrelatedness of mechanisms, there is a need for research that integrates the effects of CT across modalities. We aim to integrate (neuro)biological, psychosocial and behavioral mechanisms of CT in health and across mental and somatic disorders. The multimodal impact of CT requires more recognition in research and clinical practice and should be considered independent of current health status and diagnostic categories. Additionally, research should incorporate the impact of (daily life) stress to provide a more comprehensive understanding of the impact of CT. These recommendations may improve understanding, treatment and eventually prevention of CT-related health problems.

## Highlights

• We highlight the implications of childhood trauma for mental and somatic health.
• We integrate biological, psychosocial and behavioral mechanisms of childhood trauma.
• Interconnectedness between these modalities should guide research and treatment.
• The impact of childhood trauma should be studied and treated across diagnoses.

## 1 Introduction

Exposure to childhood trauma (CT) often has lasting effects on both mental and somatic health. Excessive stress during childhood may alter the development of the stress response system, impairing the ability to respond adaptively to stress across the lifespan (Lupien et al., 2009; Daskalakis et al., 2013; van Bodegom et al., 2017; Agorastos et al., 2018; Roberts and Lopez-Duran, 2019). CT is linked to a range of disorders, including psychosis (Varese et al., 2012), bipolar disorder (Palmier-Claus et al., 2016), alcohol dependence (Brady and Back, 2012), eating disorders (Molendijk et al., 2017), cardiovascular disease (Suglia et al., 2018) and diabetes (Widom et al., 2012). This presents a major public health concern, with global prevalence rates of childhood abuse and neglect ranging from 6.6 % to 47.2 %. The highest rates were observed in Africa for physical abuse (girls: 50.8 %, boys: 60.2 %) and in South America for neglect (girls: 54.8 %, boys: 56.7 %), while North America showed high rates for neglect (girls: 40.5 %, boys: 16.6 %) and Europe for physical abuse (girls: 12.0 %, boys: 27.0 %). These numbers are likely underestimates due to stigma and fear (Moody et al., 2018).

CT is a broad and inconsistently defined concept. For instance, the Childhood Trauma Questionnaire (Bernstein et al., 2003) defines CT as emotional, physical, or sexual abuse or emotional or physical neglect before the age of 18, while other instruments include additional stressors like bullying, poverty, parental separation or natural disasters (Bifulco et al., 1994; Breidenstine et al., 2011; Goldberg and Freyd, 2006; Smith et al., 2002; Wolfe et al., 1996). CT is often used interchangeably with terms like early life stress, which is common in animal research, and childhood maltreatment, which captures similar events but not trauma explicitly. Another term, adverse childhood experiences (ACEs), stems from a landmark study on the long-term effects of childhood abuse, neglect and household dysfunction (Felitti et al., 1998). A key limitation of this broader definition is the assumption that all ACEs equally affect health, whereas mental health outcomes are primarily driven by abuse and neglect (Negriff, 2020; Lee et al., 2020). Although these definitions may share underlying mechanisms, precision and consistency are essential for studying CT. We therefore adopt the common operational definition of CT as emotional, physical, and sexual abuse, and emotional and physical neglect before age 18.

Despite the well-established relationship between CT and mental and somatic disorders, evidence-based interventions targeting CT-related mechanisms are lacking. This gap is critical, as CT may significantly affect the course of these disorders. For example, depression in individuals with CT emerges earlier with more severe and recurrent symptoms (Teicher and Samson, 2013; Nanni et al., 2012), increased levels of anxiety, suicidality and insomnia (Hovens et al., 2012; Miniati et al., 2010) and higher comorbidity with cardiovascular disorders (Souama et al., 2023). Although treatment outcomes for major depressive disorder appear comparable for individuals with and without CT, residual symptoms are more common in those with CT due to greater baseline severity (Kuzminskaite et al., 2022). Similarly, CT is linked to earlier onset and greater severity of bipolar disorder (Quidé et al., 2020) and psychosis (Stanton et al., 2020). A meta-analysis of quasi-experimental studies supports a causal role of CT in various mental health disorders (Baldwin et al., 2023). In addition, CT subtypes may differentially influence psychiatric outcomes, further underscoring the complexity of these associations (Carr et al., 2013). CT also increases the risk for somatic health problems, including neurological, musculoskeletal and respiratory problems, cardiovascular disease, gastrointestinal, metabolic and autoimmune disorders (Wegman and Stetler, 2009; Goodwin and Stein, 2004). The underlying (neuro)biological and psychosocial mechanisms are likely interconnected and may explain variability in health outcomes. Rather than a one-to-one relationship, CT may exert many-to-many effects, where type, severity, chronicity, and timing of trauma, as well as resilience factors, influence multiple (neuro)biological and psychosocial systems, which may in turn be linked to numerous health problems. These findings underscore the need to better understand the broad impact of CT across disorders to develop more effective treatments.

To fully understand the impact of CT, research should examine its influence across interconnected biological and psychological domains. Previously, Hakamata et al. (2022) reviewed the impact of ACEs on the hypothalamic–pituitary–adrenal (HPA) axis, immune function, brain structure and function, and (epi)genetics in individuals with and without mental disorders. McLaughlin et al. (2020) proposed a risk and resilience model for CT involving interpersonal violence, focusing on psychopathology. They highlighted emotional and social information processing and accelerated biological aging as key transdiagnostic targets for early intervention. We aim to expand on these findings by I) integrating possible (neuro)biological, psychosocial, and behavioral mechanisms of CT, II) examining its impact across mental and somatic disorders, and III) emphasizing the importance of and adhering to a precise, consistent definition of CT, as outlined earlier. Rather than providing an in-depth review of any single modality, our goal is to offer a broad, integrative perspective that synthesizes the multifaceted nature of CT across disciplines. First, we highlight the implications of CT for mental and somatic health (care), including current treatment strategies and outcomes. Second, we evaluate potential mechanisms of CT across various modalities. Finally, we integrate our findings and argue that the multimodal impact of CT requires increased recognition as a starting point for understanding, treating and preventing complaints across mental and somatic disorders.

## 2 Clinical implications of CT

**Fig. 1:**
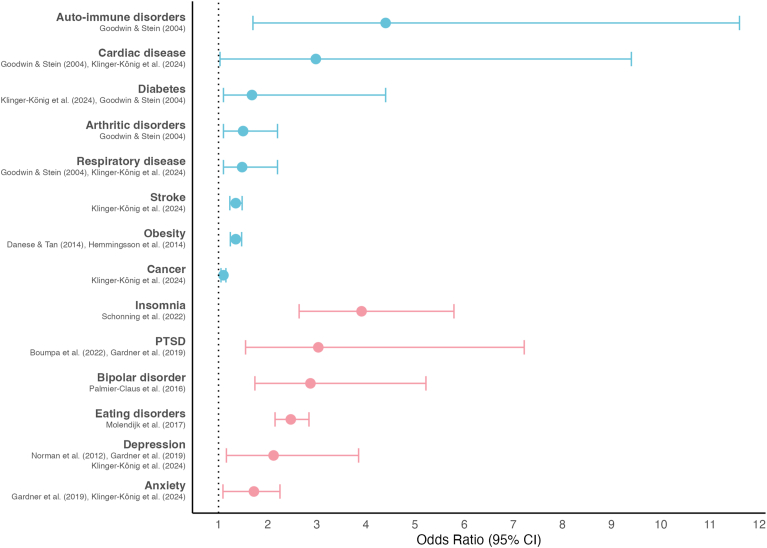
**Broad impact of CT on mental and somatic health**. This figure depicts odds-ratios (ORs) from literature discussed in the review for illustrative purposes. Blue represents somatic disorders; pink represents mental disorders or complaints. The dots represent ORs, while the error bars represent confidence intervals. ORs are averaged where multiple studies are listed. In those cases, the minimum and maximum confidence intervals are applied. Note, caution is warranted when comparing these ORs, due to differences in (meta-analytical) methods and CT subtypes. In addition, ORs depicted here are not exhaustive. PTSD = post-traumatic stress disorder; CI = confidence interval.

CT has far-reaching consequences for long-term health, underscoring the need for a deeper understanding of its clinical impact. This section outlines the implications of CT for both mental and somatic health care and emphasizes the need for targeted treatment strategies by reviewing current treatment outcomes. An overview of CT's broad health impact is provided in Fig. 1.

### 2.1 Clinical implications in mental health(care)

CT is a well-established risk factor for the onset and poorer course of numerous mental disorders in both adolescence and adulthood. Specifically, meta-analytic evidence indicates that individuals with prospectively reported CT exposure were nearly twice as likely (odds ratio; OR = 1.55, 1.69, and 1.74 for neglect, physical abuse, and emotional abuse, respectively) to develop mental disorders in adulthood compared to their non-maltreated peers (McKay et al., 2022). Accordingly, high rates of CT are commonly observed in those with mental disorders. For instance, 46–62 % of adults with major depressive disorder (MDD) report a history of CT, with approximately 19 % reporting multiple subtypes (Kuzminskaite et al., 2022; Nelson et al., 2017). High rates of CT are also found in individuals with opioid use disorders (38–43 %), eating disorders (21–59 %), and personality disorders, particularly borderline personality disorder (25–90 %) (Cattane et al., 2017; Molendijk et al., 2017; Porter et al., 2020; Santo et al., 2021). Emotional abuse and neglect are the most frequently reported subtypes across these conditions (Molendijk et al., 2017; Nelson et al., 2017; Santo et al., 2021; Zashchirinskaia and Isagulova, 2023).

Meta-analyses consistently link CT to greater symptom severity, chronicity and comorbidity of mental disorders, as well as increased suicidality (Agnew-Blais and Danese, 2016; Bailey et al., 2018; Kuzminskaite et al., 2022; Molendijk et al., 2017; Nanni et al., 2012; Nelson et al., 2017; Trotta et al., 2015). For instance, individuals with self-reported CT are approximately twice as likely to experience recurrent depression, psychotic symptoms (Nanni et al., 2012; Trotta et al., 2015), and two to three times as likely to show suicidal or self-harming behavior (Agnew-Blais and Danese, 2016; Angelakis et al., 2019; Molendijk et al., 2017; Norman et al., 2012). A two to four-fold increased risk of comorbid mental disorders (e.g., post-traumatic stress disorder (PTSD), substance/alcohol misuse disorder, anxiety, depressive disorder) in the context of eating disorders and affective disorders was found in individuals with CT (Agnew-Blais and Danese, 2016; Hovens et al., 2010; Molendijk et al., 2017). Moreover, CT has been linked to more severe obsessive-compulsive symptoms (Destree et al., 2021; Ou et al., 2020), higher prevalence of PTSD and bipolar disorder (Boumpa et al., 2022; Gardner et al., 2019; Palmier-Claus et al., 2016), and sleep problems, such as lower sleep duration and more severe insomnia symptoms (Schonning et al., 2022). Individuals with self-reported CT also show an earlier age of mental disorder onset, with roughly four years earlier onset of depression (Agnew-Blais and Danese, 2016; Molendijk et al., 2017; Nelson et al., 2017).

Differential effects of CT subtypes have also been reported. Specifically, a meta-analysis of 44 studies found that physical abuse, sexual abuse, and unspecified neglect were most strongly associated with mood and anxiety disorders; emotional abuse most strongly correlated to personality disorders and schizophrenia; and physical neglect was most related to personality disorders, though this link was weakest (Carr et al., 2013). However, trauma types frequently co-occur, and most adverse outcomes are often seen in severe CT cases, including multiple repetitive CT incidents or types (Angelakis et al., 2019; Destree et al., 2021; Hovens et al., 2010; McKay et al., 2022). For instance, combined abuse and neglect has been associated with an increased risk of MDD and bipolar disorder, exceeding additive effects (Alkema et al., 2024). Moreover, dose-dependent relationships have been identified for the number of CT types and PTSD symptomatology and poor mental quality of life (Agorastos et al., 2014). Accordingly, assessing both the distinct effects of individual CT subtypes and their cumulative impact is essential to understanding the relationship between CT and mental health outcomes.

Although CT is a common and potent risk factor for poor mental health outcomes, there are currently no treatment strategies specifically developed to target CT outside the context of PTSD and personality disorders. Meta-analytic evidence on the impact of CT on psychiatric treatment outcomes remains limited. Available studies suggest that individuals with CT show lower response and remission rates to treatment for depression (OR = 1.43) (Nanni et al., 2012; Nelson et al., 2017) and poorer outcomes in psychotic disorders, including less improvement in symptoms and social or occupational functioning (OR = 1.51) following psychotherapy or pharmacotherapy (Thomas et al., 2019). However, more recent findings offer a more nuanced perspective. A recent meta-analysis revealed that while depressed adults with CT exhibit higher symptom severity at the start and end of treatment, they benefit comparably from first-line depression treatments relative to those without CT, with similar dropout rates (Kuzminskaite et al., 2022). Additionally, psychological interventions appear effective in reducing a range of symptoms in individuals with CT. For instance, various psychotherapies, including cognitive-behavioral, insight-oriented, eclectic, and others, have been shown to alleviate trauma-related symptoms, internalizing and externalizing symptoms, self-esteem and global functioning in adults with a history of childhood sexual abuse (Taylor and Harvey, 2010). Trauma-focused therapies, such as trauma-focused cognitive behavioral therapy and eye movement desensitization and reprocessing (EMDR), are particularly effective in reducing PTSD symptoms following CT, with individual treatments outperforming group formats (Ehring et al., 2014). Accordingly, EMDR was found to be more efficacious in reducing PTSD, depressive, and anxiety symptoms than other active (cognitive behavioral therapy, individual/group therapy, fluoxetine) or control treatments (pill placebo, active listening, EMDR delayed treatment, care-as-usual) in children and adults with CT (Chen et al., 2018). Although existing treatments may benefit patients with CT in reducing various symptomatology, treatment effects are only small-to-moderate and increased severity of symptoms as well as worse illness course in individuals with CT warrants further attention to develop personalized (preventative) interventions.

In conclusion, CT is a significant risk factor for the onset, severity, and chronicity of various mental disorders. Despite its well-documented impact, evidence-based interventions specifically addressing CT-related mental health outcomes remain limited. These findings underscore the urgent need for future research to develop and evaluate personalized treatment approaches tailored to the specific characteristics and consequences of CT.

### 2.2 Clinical implications in somatic health(care)

CT has been widely associated with poorer somatic health outcomes. For instance, the relationship between CT and obesity has been well-established. Two meta-analyses, one including 41 studies (n = 190,285) and another with 23 cohort studies (n = 112,708), found a similar increased risk of adult obesity following CT, with odds ratios of 1.36 and 1.34 respectively (Danese and Tan, 2014; Hemmingsson et al., 2014). These associations were consistent across definitions of CT and obesity and across study designs (prospective vs. retrospective), highlighting the robustness of the findings. CT has also been linked to other cardiometabolic risk factors. Cross-sectional studies report higher rates of dyslipidemia (Kisely et al., 2023) and metabolic syndrome (Lee et al., 2014) among adults exposed to CT. A recent 9-year longitudinal study further supports these findings, showing that individuals with a history of CT have a consistently poorer metabolic profile over time compared to those without such exposure (Souama et al., 2023).

CT has also been associated with neurological, musculoskeletal and respiratory problems, cardiovascular disease, gastrointestinal and autoimmune disorders, and arthritic conditions (Wegman and Stetler, 2009; Goodwin and Stein, 2004). While CT subtypes often co-occur, some may be more strongly linked to such diseases than others. For instance, physical abuse was specifically associated with an increased risk of respiratory diseases (OR = 1.5) and arthritic disorders (OR = 1.5), while sexual abuse was linked to an increased risk of cardiac diseases (OR = 3.7), and neglect to diabetes (OR = 2.2) and autoimmune disorders (OR = 4.4) (Goodwin and Stein, 2004). In addition, a history of multiple CT subtypes has been associated with overall poorer physical health-related quality of life (Agorastos et al., 2014).

As research has primarily focused on mental health outcomes following CT, the relative risk of poorer somatic health compared to mental health following CT remains unclear. A meta-analysis of 24 studies (n = 48,801) reported a small-to-medium effect size (d = 0.42) for overall poorer physical health following CT, comparable to effect sizes reported for mental health outcomes (Wegman and Stetler, 2009). However, a recent study directly comparing somatic and mental health risks following CT within the same sample (n = 156,807) found higher odds for mental disorders (depression OR = 2.36, anxiety OR = 2.08) than for somatic conditions such as cancer (OR = 1.10), myocardial infarction (OR = 1.13), diabetes (OR = 1.16), stroke (OR = 1.35), and chronic obstructive pulmonary disease (OR = 1.45) (Klinger-König et al., 2024). Importantly, CT has also been associated with comorbid physical and mental illness. For example, individuals who experienced CT were found to be three times as likely to have comorbid cardiometabolic disease and depression (OR = 3.04) (Souama et al., 2023). These findings underscore the relevance of CT as a shared risk factor linking mental and somatic health.

Research on the role of CT in overall health has primarily focused on mental health, and clinical guidelines for mitigating its impact on somatic health are lacking. For instance, despite the well-established relationship between CT and obesity, it is unclear if and how clinical interventions could mitigate the developmental predisposition to obesity in individuals with CT (Danese and Tan, 2014). As a starting point, increased awareness of the role of CT in somatic health may facilitate in-depth monitoring of somatic health in those with a history of CT to support the implementation of early interventions and preventive strategies, such as lifestyle adjustments and medication (Souama et al., 2023).

## 3 Molecular mechanisms

A vast array of literature describes the relation between CT and several molecular mechanisms. Here, we highlight the link between CT and disturbances in the HPA-axis, the immune system, (epi)genetics, and biological aging. We also discuss potential mechanisms of action underlying these associations.

### 3.1 The HPA-axis

By definition, CT occurs during a critical developmental period characterized by elevated neuroplasticity and may therefore affect stress system development (Agorastos et al., 2018). These effects are mediated by the hypothalamic-pituitary-adrenal (HPA)-axis, the major neuroendocrine system regulating the body's response to stress, including its end product cortisol. CT, accompanied by high cortisol levels and glucocorticoid receptor (GR) overactivation, may ultimately cause altered development of the stress system (Daskalakis et al., 2013). While this response may be adaptive in the short-term (i.e., lower threshold of detecting threats), it may come at the cost of long-term maladaptation: a reduced capacity to adequately and dynamically respond to stress across the life span. Accordingly, CT has been consistently linked to altered HPA-axis functioning in adulthood, including both its basal and stress-induced activity (Heim et al., 2008; Bunea et al., 2017; Murphy et al., 2022). Both increased and blunted cortisol responses to acute stress have been reported following CT (Bunea et al., 2017; Murphy et al., 2022). These seemingly contradictory findings may be attributed to characteristics of CT, such as the type, duration, intensity and number of exposures, as well as personal characteristics, such as age (i.e., developmental stage), sex, or individual vulnerability (e.g., genetic or epigenetic susceptibility) (Agorastos et al., 2018). Dependent on these factors, CT may cause sustained overactivation on hodological associations of the HPA-axis, such as the hippocampus, amygdala, and medial prefrontal cortex (mPFC) (Murphy et al., 2022).

Importantly, similar HPA-axis alterations following CT have been observed across different mental disorders. For instance, Murphy et al. (2022) identified a shared HPA-axis profile in bipolar disorder and psychosis, but not depression, characterized by increased long-term cortisol and cortisol turnover and attenuated cortisol responses to awakening, stress and physiological manipulation following CT. These alterations align with structural abnormalities in stress-related brain circuits common to both disorders. Together, this suggests that CT-related HPA-axis dysregulation may increase vulnerability to mental illness beyond diagnostic boundaries.

HPA-axis alterations following CT remain understudied in the context of somatic disorders, despite clear evidence linking CT to lasting HPA-axis changes. This is a critical gap, as dysregulated HPA-axis function increases the risk for conditions such as cardiovascular, metabolic, and endocrine disorders (Kadmiel and Cidlowski, 2013).

### 3.2 The immune system

CT has also been consistently linked to both basal and stress-induced immune system alteration. Individuals with CT histories exhibit greater inflammatory responses to both daily and experimental stressors (Carpenter et al., 2010; Gouin et al., 2012; Kiecolt-Glaser et al., 2011; Pace et al., 2006). Additionally, elevated basal inflammation levels were found in depressed individuals with CT compared to those without, although some findings were compromised by low statistical power (Danese et al., 2008). A large meta-analysis by Baumeister et al. (2016) confirmed associations between CT and increased peripheral levels of C-reactive protein (CRP), interleukin-6 (IL-6), and tumor necrosis factor alpha (TNF-α). While age, sex, and body mass index (BMI) did not moderate these effects, trauma subtype did; sexual abuse was more strongly linked to TNF-α, and physical abuse to both TNF-α and IL-6. The findings were in line with another meta-analysis, showing elevated TNF-α, IL-6, and especially CRP in individuals with CT (Kuhlman et al., 2020). Furthermore, the impact of CT on pro-inflammatory markers appeared to be transdiagnostic, as shown by a recent meta-analysis including patients with mood disorders, substance use disorders, schizophrenia spectrum disorders, eating disorders, and anxiety disorders (van den Noortgate et al., 2025).

While human studies support a link between CT and immune system alterations, insights into the underlying molecular mechanisms largely come from animal research. Danese and Lewis (2017) reviewed several pathways by which early-life immune activation may lead to heightened innate immune responses later in life. One mechanism involves microglial priming: early immune activation can induce lasting changes in morphology and cell surface antigens in microglia, increasing their sensitivity to later inflammatory stimuli. Rodents with ‘primed’ microglia show greater neuroinflammation, neurotoxicity, and sickness behavior compared to those with ‘naive’ microglia (Perry and Holmes, 2014). Another mechanism is neuroendocrine cross-sensitization: inflammatory cytokines IL-1, and IL-6, and TNF-α to a lesser extent, can activate the HPA-axis and central catecholamines, mimicking the effects of psychosocial stress (Besedovsky et al., 1986; Dunn, 2000). Accordingly, neonatal immune stimulation in rodents has been shown to produce long-term effects on the HPA-axis, including long-term elevations in plasma corticosterone levels and increased lymphocyte sensitivity to stress-induced suppression of proliferation (Shanks et al., 2000). Together, these animal studies suggest that early-life immune activation may program lasting immune and neuroendocrine dysregulation, helping to explain the link between immune changes and CT observed in humans.

### 3.3 Biological aging

Another molecular mechanism commonly linked to mental and somatic health problems following CT is accelerated biological aging. Biomarkers of cumulative cellular stress, such as telomere length and mitochondrial DNA copy number, have been associated with poor mental and somatic health outcomes following neglect, and may even play a role in the intergenerational transmission of trauma (Ridout et al., 2018). However, this link was not significant for abuse. In contrast, a large meta-analysis found that early-life adversity involving threat (i.e., abuse), but not deprivation (i.e., neglect) was associated with earlier pubertal timing (n = 114,450) and greater cellular aging (n = 1560), measured via leukocyte telomere length and DNA methylation age. Similarly, children exposed to threat showed accelerated cortical thinning in the ventromedial prefrontal cortex (vmPFC), whereas deprivation was linked to thinning in the inferior frontal gyrus and areas of the frontoparietal, default-mode and visual networks (Colich et al., 2020). Despite some inconsistencies across CT subtypes, CT exposure generally appears to be associated with accelerated biological aging.

### 3.4 (Epi)genetics

Extensive research has explored the complex relation between genetics and CT, particularly whether its occurrence and effects are environmental, genetic, or both. Evidence from sibling and twin studies supports an environmental contribution. Baldwin et al. (2023) found that while genetic and shared environmental factors partly explained the association, CT independently contributed to the risk of developing psychopathology. In the same vein, discordant monozygotic twin studies found greater psychotic complaints in twins exposed to CT than their non-exposed co-twins (Alemany et al., 2013). At the same time, genetic studies highlight heritable influences. A genome-wide association study (GWAS) meta-analysis (n > 185,000) reported modest heritability for CT and significant genetic correlations with depression, schizophrenia, and ADHD (Warrier et al., 2021). Mendelian randomization further suggested both unidirectional and bidirectional causal relationships between CT and these conditions, underscoring gene-environment (GxE) interplay. In line with this, a systematic review of GxE studies found that while childhood adversity, including abuse and neglect, increased psychopathological risk regardless of genotype, specific genetic profiles markedly amplified vulnerability to adverse outcomes (Maglione et al., 2018). Similarly, this interplay was found for intergenerational effects. Maternal CT was linked to increased psychopathology in offspring, partly mediated by maternal depression (Su, D'Arcy and Meng, 2022), further suggesting that both inherited and environmental factors shape risk across generations. However, a meta-analysis of 5765 individuals found no evidence for an interaction between polygenic risk for depression and CT, suggesting that previous reports of such interactions may have been chance findings or driven by other types of adversities (Peyrot et al., 2018). Despite these advances, challenges remain in fully mapping gene-environment interplay, as genetic liability may shape both exposure risk and vulnerability to subsequent pathology. In addition, inconsistencies in findings highlight the importance of more harmonized GxE studies in large, deeply-phenotyped, longitudinal cohorts (Martins et al., 2022). This underscores the need for continued integration of genetically informed methodologies in CT research.

Additionally, the long-lasting impact of CT might be partially explained through changes in epigenetics – modifications determining whether a gene is expressed. DNA methylation, the most extensively studied epigenetic mechanism in this context, remains responsive to environmental stimuli beyond prenatal development. CT has been associated with altered DNA methylation in genes related to various biological domains, including the HPA-axis, brain-derived neurotropic factor, neurotransmitters, transcription factors (e.g., orthodenticle homeobox 2 [OTX2]), and myelination (Brown et al., 2019). These associations were further supported by a meta-analysis of five epigenome-wide association studies (EWASs), which identified 44 differentially methylated CpG sites linked to CT (Neves et al., 2021).

Importantly, CT-induced changes in epigenetics have been linked to alterations in other molecular domains. For instance, although evidence in humans is limited, rodent studies suggest that CT can induce cell type-specific epigenetic modifications, such as in astrocyte-expressed genes involved in immune signaling (Rahman and McGowan, 2022). Both animal and human research also indicate that epigenetic changes may contribute to altered basal and stress-induced HPA-axis activity following CT (Murgatroyd and Spengler, 2011). Moreover, CT was associated with accelerated epigenetic aging, an indicator of biological aging based on DNA methylation patterns (Palma-Gudiel et al., 2020). Emerging evidence further implicates epigenetic regulation of GR expression, brain development, and subsequent mental health outcomes (Nie et al., 2022).

A growing body of research indicates that CT can epigenetically influence key molecular pathways, including those involved in immune function and biological aging. These molecular changes may, in turn, affect broader biological systems such as brain networks underlying emotion, cognition, and behavior, thereby increasing vulnerability to psychiatric and somatic disorders. Nonetheless, the heterogeneity and complexity of CT-related epigenetic alterations likely contribute to the limited clinical progress in developing reliable biomarkers and targeted epigenetic therapies.

## 4 Neurobiological mechanisms

Although the neurobiological mechanisms linking CT to increased risk for mental and somatic disorders are not yet fully understood, both structural and functional brain alterations have been reported. Exposure to adversity, such as abuse and neglect, during sensitive developmental periods may shift developmental timing, particularly for brain regions with a high density of GRs, including the hippocampus, amygdala and prefrontal cortex (PFC) (Holz et al., 2023). In this section, we summarize key findings and consider individual and methodological factors that may contribute to the variability in results.

### 4.1 Brain volume and microstructure

Reduced grey matter volume (GMV) of the hippocampus, amygdala and PFC has been reported in individuals exposed to CT (Giotakos, 2020; Ahmed-Leitao et al., 2016; Woon and Hedges, 2008; Lim et al., 2014; Dahmen et al., 2018). For instance, Ahmed-Leitao et al. (2016) found bilateral reductions in hippocampal and amygdala GMV in adults with childhood maltreatment-related PTSD compared to healthy controls. These changes may develop over time, as bilateral reduction of hippocampal GMV was found in adults, but not children, with childhood maltreatment-related PTSD (Woon and Hedges, 2008), supporting the idea that traumatic experiences may negatively impact development of brain regions with high GR density. However, this study did not find any changes in amygdala GMV in either children or adults. Findings on the frontal cortex also vary, with both increases and decreases in GMV reported among individuals with CT and diagnoses including bipolar disorder, depression, schizophrenia, and anxiety (Begemann et al., 2021; Lu et al., 2019; Van Harmelen et al., 2010). Even within the PFC, regional GMV differences have been observed following CT (Richert et al., 2006; Carrion et al., 2009). These inconsistencies likely stem from differences in sample characteristics, such as CT subtype, co-morbid psychiatric disorders, age, and sex, which are not always reported. In accordance, Cassiers et al. (2018) summarized that different subtypes of CT can affect the brain distinctively. Specifically, structural deficits in the reward circuit and genitosensory cortex were related to sexual abuse. Moreover, much of the literature focuses on PTSD following CT, complicating efforts to disentangle the effects of CT from those of PTSD or their interaction.

Altered cortical thickness (CTh) and white matter integrity (WMI), have also been reported following CT. Compared to GMV, research on CTh is sparse and findings are mixed. For example, Gold et al. (2016) found that greater exposure to physical and/or sexual abuse was associated with reduced CTh in the vmPFC and bilateral parahippocampal gyrus in adolescents. In contrast, other studies report preserved CTh in the vmPFC, amygdala and hippocampus, amongst other regions, in adolescents following CT (Rinne-Albers et al., 2020; Ahmed et al., 2012). These discrepancies are likely due to methodological variability. As early as 2000, Fischl and Dale (2000) emphasized that submillimeter-accurate CTh measurements allow for more sensitive statistical analyses, yet inconsistencies in study designs still limit comparability. Nevertheless, a recent meta-analysis showed broadly overlapping reductions in CTh and GMV in children and adults with a history of CT, with the median cingulate and paracingulate gyri most consistently affected (Yang et al., 2023).

Likewise, alterations in WMI have been reported following CT, with neglect showing a particularly strong association (Cassiers et al., 2018). Hanson et al. (2013) observed more diffusely organized WMI in the PFC of children exposed to CT, which was related to neurocognitive deficits. A review and meta-analysis by Daniels et al. (2013), encompassing 25 studies in individuals with and without PTSD, found that reductions in WMI were reported more often than increases, particularly in the corpus callosum and cingulum bundle, a key white matter tract connecting the limbic system, hippocampus, and PFC. These findings are further supported by a recent meta-analysis suggesting that CT is associated with widespread reductions in WMI, most prominently in the fornix, corpus callosum and optic radiations (Lim et al., 2020). The authors suggest that these impairments may disrupt fronto-limbic and occipital connectivity, potentially interfering with the transmission and integration of (aversive) experiences.

### 4.2 Functional responsivity and connectivity

Consistent with structural findings, CT has also been associated with functional alterations in the amygdala, hippocampus, insula and dorsolateral PFC (dlPFC) (Hakamata et al., 2022; Holz et al., 2023), particularly with heightened amygdala responsivity to negative emotional stimuli (Teicher et al., 2016; Hakamata et al., 2022). These regions are part of the fronto-limbic circuit, involved in emotion and threat processing and threat generalization (Lange et al., 2019). CT may impair top-down regulation of lower-order emotional processing in the amygdala (van Harmelen et al., 2013) though findings on amygdala-mPFC connectivity remain mixed, potentially due to age or task-related differences (Holz et al., 2023). Nevertheless, aberrant fronto-limbic connectivity has been consistently observed in healthy children, adolescents, and adults, as well as in patients with MDD and PTSD exposed to CT and other childhood adversities (e.g., low socioeconomic status, bullying, illness) (Hakamata et al., 2022). Additionally, CT is linked to blunted striatal activation in response to anticipated or received reward (Teicher et al., 2016). Some studies report subtype-specific associations, such as amygdala hyperresponsivity in individuals exposed to sexual abuse, particularly during sad autobiographic memory recall, and abnormalities in fronto-limbic activity and connectivity following emotional maltreatment (Cassiers et al., 2018). These alterations in emotion and reward processing circuits may contribute to increased vulnerability to mental disorders (Teicher et al., 2016). Notably, similar patterns in clinical and non-clinical populations suggest that CT-related changes may precede disorder onset (Hakamata et al., 2022).

Additionally, recent studies have examined the effects of CT on distributed brain networks (i.e., collections of brain regions that are closely interrelated), primarily the frontoparietal (FPN) and default mode network (DMN) (Holz et al., 2023). Dynamics of these stress-related brain networks can be studied during resting-state scans, without requiring an explicit task, by analyzing changes in functional connectivity over time. This approach may reflect the general adaptability (i.e., the ability to switch between functional states and adapt to environmental demands) of functional brain networks, hence informing how the brain would adapt to a stressor. Findings suggest that individuals with a history of CT show disrupted brain dynamics during rest (Cisler, 2017; Ross et al., 2021). These disruptions may underlie impaired processing of stress and task-related information. However, more research is needed to clarify the relationship between CT and dynamic functional connectivity. Overall, current evidence indicates that CT may significantly affect cognitive-emotional processing through alterations in large-scale brain network organization (Ross et al., 2021).

In summary, CT has been associated with structural and functional brain alterations. Structural changes include reduced GMV in the hippocampus, amygdala, and PFC, as well as alterations in WMI and CTh. Functionally, CT is linked to increased stress sensitivity and disrupted emotional processing, particularly in the fronto-limbic circuit, which is involved in emotion regulation and threat response. Variability in these effects may further be influenced by trauma type and co-occurring psychiatric conditions, highlighting the importance of taking these factors into account. Together, these structural and functional alterations may heighten an individual's susceptibility to stress, potentially leading to adverse health outcomes. Notably, most research to date has focused on the fronto-limbic circuit. Future studies should also examine large-scale brain networks and their interactions to provide a more comprehensive understanding of the neurobiological impact of CT.

## 5 Psychosocial mechanisms

Psychosocial behavior also influences the relationship between CT and general health. Previous systematic reviews have identified attachment style, early maladaptive schemas, maladaptive personality traits, self-esteem, emotion regulation, coping, and perceived social support as potential transdiagnostic mechanisms (Panagou and MacBeth, 2022; Hoppen and Chalder, 2018; Kuzminskaite et al., 2021; McLaughlin et al., 2020). These mechanisms are discussed below.

### 5.1 Attachment style

Attachment theory focuses on (long-term) relationships between people, particularly between parent and child. Attachment can be conceptualized as a psychobiological system that drives individuals to seek security, comfort, and closeness to significant others in times of need (Bowlby, 1969). CT can disrupt attachment to primary caregivers (Toof et al., 2020; Baer and Martinez, 2006), often resulting in insecure attachment styles, such as anxious and avoidant attachment. Individuals with attachment anxiety tend to view themselves as unworthy, incompetent, and powerless, strongly desire intimacy in relationships, and fear rejection and abandonment (Diamond and Fagundes, 2010). In contrast, those with avoidant attachment are less comfortable with intimacy and tend to avoid investing in emotional relationships due to expectations of unsupportive responses (Diamond and Fagundes, 2010). Insecure attachment has been linked to increased vulnerability to later trauma and psychopathology (Breidenstine et al., 2011). Accordingly, emotional abuse and neglect in childhood were associated with greater attachment anxiety in a non-clinical adolescent sample (n = 449; Falgares et al., 2024). Similarly, attachment anxiety mediated the relationship between childhood emotional neglect and anxiety and depression in late life in a cross-sectional study of older adults (n = 81; Van Assche et al., 2020). Avoidant attachment in individuals with CT has also been associated with elevated suicide risk (Ihme et al., 2022). As attachment styles can influence the client-therapist relationship, and therefore therapy outcomes, assessing attachment style at the start of treatment may be beneficial.

### 5.2 Core beliefs and early maladaptive schemas

Attachment is closely related to the way core psychological needs (i.e., safety, autonomy, emotional support, freedom to express needs and emotions) are met in childhood. When these needs are unmet, negative core beliefs or early maladaptive schemas may develop, shaping the way individuals respond to others and stressful events throughout life (Young et al., 2003). Schemas incorporate beliefs about the self, others, and the world, and greatly influence someone's identity, their expectations about life, and the quality of their relationships. Meta-analytic findings suggest that CT, particularly emotional abuse and neglect, is associated with early maladaptive schemas in adolescence (May et al., 2022). Specifically, emotional abuse was linked to “emotional deprivation” (the belief that the need for emotional support will not be met) and “subjugation” (excessive surrendering of control, typically to avoid conflict or abandonment). Emotional neglect was associated with schemas such as “mistrust/abuse” (expectation of harm or exploitation by others), “abandonment” (perceived instability or unreliability of those available for support and connection), “social isolation” (feeling disconnected or different from others) and “failure” (belief of failure or fundamental inadequacy).

Similar patterns have been observed in adults, as evidenced by a meta-analysis of 33 studies reporting associations between CT and maladaptive schemas later in life (Pilkington et al., 2021). Emotional neglect was again associated with the schemas “failure” and “emotional deprivation”, while emotional abuse was associated with “emotional deprivation” and “vulnerability to harm” (i.e., an exaggerated fear of imminent catastrophe and helplessness to prevent it). Smaller associations were found between physical neglect, physical abuse and sexual abuse and “vulnerability to harm”, “emotional deprivation” and “social isolation”. However, data on some adversities, including sexual abuse and neglect, were limited, highlighting the need for further research.

In sum, early maladaptive schemas, shaped by unmet psychological needs following CT, are associated with later psychopathology in both children and adults (Bishop et al., 2022; Maher et al., 2022; Hawke and Provencher, 2011; Barazandeh et al., 2016; Nicol et al., 2020), suggesting they may mediate the relationship between CT and mental health outcomes.

### 5.3 Personality traits

Early maladaptive schemas and negative core beliefs may become deeply rooted in an individual's personality. In fact, personality traits are also considered possible transdiagnostic factors underlying the association between CT and health outcomes. An often cross-culturally used model to describe personality is the five-factor model, which encompasses five personality traits: extraversion, agreeableness, openness, conscientiousness, and neuroticism (Goldberg, 1990). Although no systematic reviews or meta-analyses have specifically examined the link between CT and these traits, several large-scale studies consistently point to a significant association. For instance, in the Netherlands Study of Depression and Anxiety (NESDA) cohort study on the development and long-term prognosis of anxiety and depression in adults (n = 2974), CT (emotional neglect and abuse in particular) was associated with higher levels of neuroticism and openness and lower levels of extraversion, conscientiousness and agreeableness (Spinhoven et al., 2016). Similarly, a self-report study in 1116 adults found that CT was positively associated with neuroticism. For other personality traits, subtype-specific associations were observed: emotional abuse correlated with higher openness and agreeableness, sexual abuse with higher agreeableness, and emotional neglect with lower conscientiousness, extraversion, and agreeableness (Alnassar et al., 2024). A similar association was found in patients with schizophrenia (n = 374), whereby CT was associated with increased neuroticism and decreased agreeableness and conscientiousness (Adanty et al., 2022). Maladaptive personality traits, particularly neuroticism, may play a key role in the link between CT and psychopathology. Neuroticism has been shown to significantly mediate the relationship between emotional abuse and symptoms of depression and anxiety (Alnassar et al., 2024), as well as between CT and depression severity in individuals with bipolar disorder (Wrobel et al., 2022). The alternative model for personality disorders of the fifth Diagnostic and Statistical Manual of Mental Disorders (DSM-5) includes five trait domains to describe pathological personality characteristics: negative affectivity, detachment, antagonism, disinhibition, and psychoticism (American Psychiatric Association, 2013). A narrative review on this personality model has indicated that (mainly emotional) CT is especially associated with negative affectivity (i.e., frequent and intense experiences of negative emotions), detachment (i.e., avoidance of socioemotional experience) and psychoticism (i.e., culturally incongruent odd, eccentric, or unusual behaviors and cognitions) (Back et al., 2021). In addition, findings from a cross-sectional study indicate that negative affectivity and detachment might mediate the effect of childhood sexual abuse on internalizing psychopathology in adulthood (Veith et al., 2017).

### 5.4 Self-esteem

CT, including both abuse and neglect, has been consistently linked to lowered self-esteem in children and adults (Zhang et al., 2023), which is proposed as a critical mechanism mediating its impact on mental health (Hoppen and Chalder, 2018; Zhao et al., 2022). Self-esteem refers to an individual's evaluation of their self-worth or personal value (Leary and Baumeister, 2000), with distinctions drawn between explicit self-esteem (conscious self-appraisal) and implicit self-esteem (unconscious associations and feelings towards the self) (Van Tuijl et al., 2016; Creemers et al., 2012; Gathier et al., 2024). Most research on the role of self-esteem in the link between CT and mental health has focused on depression and anxiety, showing that explicit self-esteem, typically measured by self-report questionnaires such as the Rosenberg self-esteem scale (Rosenberg, 1965), mediates the relationship between CT and symptom severity in children, adolescents and adults (Gathier et al., 2024; Berber Çelik and Odacı, 2020; Chen et al., 2022; Kim et al., 2022; Li et al., 2023; Reid-Russell et al., 2022; Wang et al., 2022; Yoon et al., 2019).

### 5.5 Emotion regulation, coping, and perceived social support

Emotion regulation, coping and perceived social support are additional transdiagnostic factors implicated in the relationship between CT and mental health. Meta-analyses show that CT relates to decreased emotion regulation in childhood and adolescence (Gruhn and Compas, 2020; Lavi et al., 2019) and that emotion regulation difficulties mediate the relationship between CT and psychopathology (Miu et al., 2022). Several cross-sectional studies support this the mediating role, particularly in relation to adult depression (Hopfinger et al., 2016; Huh et al., 2017), anxiety (Huh et al., 2017), borderline personality features and disorder (Peng et al., 2021), and psychotic experiences (Lincoln et al., 2017). Emotion regulation is also closely tied to how individuals manage and cope with stressful experiences (Gruhn and Compas, 2020). Meta-analytic evidence indicates that avoidant coping strategies (e.g., denial, social withdrawal and emotional disengagement) are significantly associated with both child maltreatment (Gruhn and Compas, 2020) and psychological distress following trauma (Littleton et al., 2007). In contrast, cross-sectional findings indicate that positive coping strategies (e.g., seeking social support, reframing negative thoughts, learning from others) and perceived social support may buffer the adverse effects of childhood maltreatment on mental health (Su, D'Arcy and Meng, 2020; Cao et al., 2022).

## 6 Lifestyle factors

Finally, lifestyle factors may play a critical role in the relationship between CT and mental and somatic health outcomes. Meta-analytic findings indicate that physical and emotional abuse and emotional neglect are associated with a range of unhealthy behaviors, including alcohol and drug use, smoking, risky sexual behavior, physical inactivity, and interpersonal and self-directed violence (Norman et al., 2012). A recent cohort study (n = 2968) confirmed a dose-response relationship between CT and behaviors such as smoking, drug use, social inactivity, sleep deprivation and excessive weight gain (Tong et al., 2024). These behaviors, in turn, contribute to adverse mental and somatic health outcomes. For instance, a UK biobank study (n = 110,596) found that unhealthy behaviors (e.g., smoking, poor diet, physical inactivity) mediated the relationship between CT and accelerated aging (Yang et al., 2022).

The influence of lifestyle factors on the relationship between CT and health outcomes is closely interconnected with biological and psychological mechanisms. The link between CT and substance use, abuse, and addiction illustrates this complex interplay. CT has been implicated in all stages of substance use and addiction, from initiation to dependence, relapse, and treatment response. Although substance use is typically considered a behavioral pattern, individuals with CT histories may also exhibit increased biological vulnerability to addiction. These include alterations in the HPA axis and stress-related brain systems, which may amplify the reinforcing effects of substances during initiation, maintenance, withdrawal, and relapse (Moustafa et al., 2021). Psychological factors, such as mood and impulsivity, further moderate the relationship between CT and substance abuse (al’Absi et al., 2023). Taken together, lifestyle factors likely play a critical role in the relationship between CT and mental and somatic health outcomes, yet they are deeply intertwined with biological and psychological factors, making their individual effects difficult to disentangle.

These findings highlight the importance of integrated interventions that address not only lifestyle factors, such as nutrition, physical activity, and substance use prevention, but also the underlying psychological needs driving these behaviors. Since unhealthy lifestyle choices may serve as ways to cope with stress for individuals exposed to CT (Tong et al., 2024; Yang et al., 2022), effective prevention and treatment require a comprehensive approach that considers the biological, psychological, and behavioral pathways linking CT to adverse health outcomes.

## 7 Multimodal pathways of CT

**Fig. 2:**
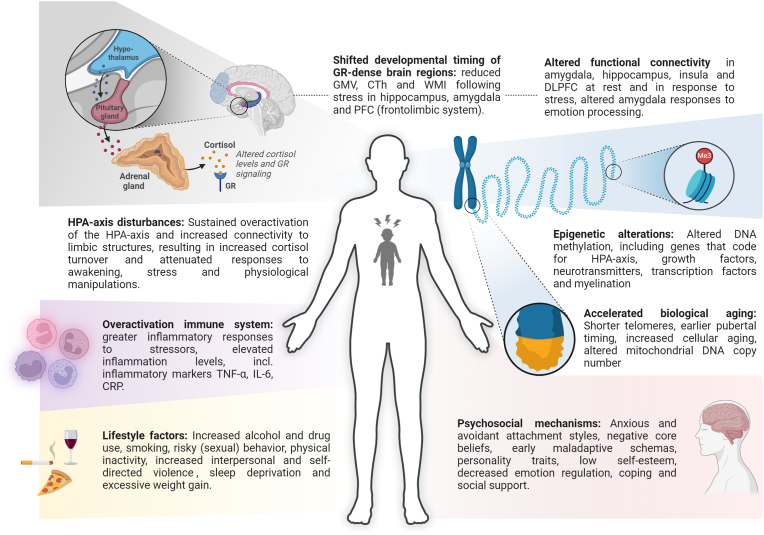
**Transdiagnostic mechanisms linking childhood trauma exposure to mental and somatic health.** Childhood trauma exerts profound, long-term impact across biological, psychological, and behavioral domains, including brain structure and function, the HPA-axis, the immune system, epigenetics, biological aging, lifestyle, and psychosocial mechanisms. GR = glucocorticoid receptor, HPA = hypothalamus-pituitary-adrenal, CT = childhood trauma, TNF-α = tumor necrosis factor alpha, IL-6 = interleukin 6, CRP = c-reactive protein, GMV = grey matter volume, CTh = cortical thickness, WMI = white matter integrity, PFC = prefrontal cortex, dlPFC = dorsal lateral prefrontal cortex, DNA = deoxyribonucleic acid. Figure created using BioRender.

Given the widespread and heterogeneous impact of CT (see Fig. 2), research should aim to integrate its effects across biological, psychological, and behavioral domains, rather than studying them in isolation. Here, we propose two hypothetical multimodal pathways explaining the lifelong effects of CT and illustrate their added value for understanding and treating its consequences. These pathways are neither mutually exclusive nor exhaustive.

First, the long-term impact of CT might be explained through a *stress sensitization pathway,* involving both hypervigilance (i.e., heightened alertness and sensitivity to potential threats) and increased stress reactivity. This might be adaptive at first, allowing children to detect and handle potential threats effectively, but maladaptive in the long-term due to altered stress system development and maladaptive personality traits. Accordingly, CT is associated with increased cortisol signaling, altered structural development and overactivation of GR-dense brain regions (i.e., the hippocampus, amygdala and PFC), amygdala over-reactivity to negative emotional stimuli and increased neuroticism, characterized by emotional instability and stress vulnerability. Concurrently, earlier pubertal timing and increased immune signaling might serve to prepare an individual to handle potential threats. Together, these physiological and psychological changes may contribute to heightened negative affect, increased subjective stress, and greater vulnerability to (psycho)pathology. As such, bridging biological, psychological, and behavioral domains is essential to fully capture the complex and long-term consequences of CT.

Second, evidence supports a *threat avoidance pathway,* whereby individuals with CT display more avoidant, dissociative, or passive behaviors. Similar to stress sensitization, avoidance may initially be an adaptive coping mechanism, helping children maintain necessary relationships with abusive caregivers and reduce harm. However, persistent avoidance often results in lower self-esteem, social isolation, impaired emotion regulation, and difficulties in relationships. Potential neurobiological factors underlying threat avoidance include altered connectivity in emotion-processing circuits, reduced cortisol responses to stress, and diminished brain reactivity to anticipated rewards. These changes are accompanied by psychosocial traits commonly seen following CT, such as detachment, lower extraversion, avoidant attachment, and subjugation. Personality traits like elevated neuroticism, also frequently associated with CT, may further amplify avoidance by increasing emotional distress and withdrawal. Despite these converging findings, the direct link between neurobiological and psychosocial components of this pathway remains understudied.

Taken together, a multimodal approach facilitates the development of more comprehensive hypotheses and a deeper understanding of overarching mechanisms, such as vigilance, stress reactivity, and threat avoidance. This approach may also account for heterogeneity in the literature, reflecting not only methodological variation, but also distinct pathways of adaptation to CT. Finally, integrating modalities provides more detailed monitoring and personalized treatment options, as individuals with a biological risk profile for one pathway may benefit from targeted psychological treatments and vice versa.

## 8 Integration, conclusions and recommendations

This review highlights key (neuro)biological, psychosocial, and behavioral pathways of CT, and integrates its impact across mental and somatic disorders. We have shown that CT is associated with changes in epigenetics, biological aging, the HPA-axis, the immune system and brain structure and function, as well as psychosocial and lifestyle factors. Despite its broad health implications, evidence-based (transdiagnostic) treatments targeting the impact of CT remain scarce. We recommend increased acknowledgement and implementation of five key facets imperative in studying and treating the impact of CT: multimodal evaluation, a transdiagnostic scope, understanding resilience, precision and consistency in CT assessment, and involving daily-life stress.

Firstly, advancing the understanding and treatment of CT requires a multimodal approach. As demonstrated in this review, the effects of CT span across modalities, ranging from epigenetic alterations to maladaptive schemas. Section seven further outlines how these modalities may be interconnected. Such a multimodal perspective is essential to disentangle vulnerability from resilience and to develop personalized treatments. To support this integration, computational psychiatry offers a promising framework by using modeling techniques to link lower-level neurobiological disruptions with higher-level behavioral and functional outcomes (Murray et al., 2018). While this emerging field has so far primarily aimed to uncover the neural basis of mental disorders, this approach would also be well-suited for linking the impact of CT across different modalities.

Secondly, studying and treating the impact of CT requires a transdiagnostic approach. This review demonstrates that molecular, (neuro)biological, psychosocial, and clinical alterations associated with CT have consistently been found across individuals with and without mental or somatic disorders and irrespective of specific diagnoses. For example, disruptions in functional connectivity of stress-related brain circuits following CT were found across individuals with MDD, PTSD, and healthy controls. Likewise, psychosocial mechanisms such as impaired emotion regulation appear to underlie the relationship between CT and various mental disorders, including anxiety, borderline and psychotic disorders. Furthermore, CT is linked to lifestyle factors, such as smoking, substance abuse and poor diet, that contribute broadly to adverse mental and somatic health outcomes. These findings emphasize the need to recognize CT as a transdiagnostic risk factor, rather than confining its relevance within traditional diagnostic categories. While we do not propose the disregard of diagnosis altogether, we do argue for CT to take more center stage. As a starting point, individuals with CT should be differentiated from those without, both within and between diagnostic categories. For instance, as suggested by Teicher et al. (2022), specifiers indicating CT history could be added to the DSM to delineate possible CT-related ecophenotypes. Currently, the DSM-5 only briefly references childhood maltreatment under “conditions that may be a focus of clinical attention but are not mental disorders” (V-codes). Although the ICD-10 does have more specific codes indicating childhood maltreatment (Z-codes), there is a lack of awareness about them. Additionally, there is currently no evidence-based guidance for clinicians on using these codes, which may explain their limited application in practice.

Thirdly, the impact of CT in those *without* diagnoses requires increased attention in research and clinical practice. Our review reveals similar (neuro)biological correlates of CT in clinical and non-clinical populations. While not all individuals with a history of CT will develop health problems, this may suggest that in some cases, CT-related disturbances may exist prior to the onset of overt pathology. Possibly, compensatory biological mechanisms, personal characteristics, or environments buffer or delay the potential adverse effects of CT. Although resilience following CT has been widely studied and conceptualized (Pasteuning et al., 2024), comprehensive longitudinal research on resilient individuals remains limited. Given the dynamic nature of resilience, individuals may be temporarily protected from ill health but remain vulnerable over time. To support longitudinal research on resilience after CT, we refer to our earlier work outlining a multilevel dynamic framework and reporting checklist that highlight its complexity and temporal nature (Pasteuning et al., 2024). In clinical practice, we recommend screening for CT history at an earlier stage, for instance when people visit their general practitioner for mental or somatic complaints.

Fourthly, precision and consistency in assessing CT are essential to improve the comparability and interpretation of findings. The term CT is often used interchangeably with broader constructs such as ACEs, which include additional stressors like poverty and parental separation. Moreover, many studies fail to report a clear definition. Our findings, showing differential effects of CT subtypes and severity on health outcomes, highlight how this lack of clarity hinders progress in the field. For example, childhood abuse and neglect likely have different impacts than less threatening stressors, such as parental separation. Moreover, the developmental timing of CT exposure plays a critical role in shaping mental and somatic health outcomes. Agorastos et al. (2018, 2019) emphasize the impact of CT during periods of heightened neuroplasticity on stress system development and related neurobiological pathways. In line with this, our findings reveal distinct (micro)structural and functional alterations in fronto-limbic brain regions in individuals with CT. Consequently, clearer consensus on CT definitions along with explicit reporting of subtypes, duration, timing, and chronicity is essential for both research and clinical practice. The 52-item Maltreatment and Abuse Chronology of Exposure (MACE) scale offers a valuable retrospective tool that captures these factors by assessing the timing, chronicity, and severity of various maltreatment subtypes year by year during childhood (Teicher and Parigger, 2015).

Finally, understanding the impact of CT requires more focus on daily life stress. Individuals with a history of CT show altered HPA-axis reactivity and inflammatory responses to acute stress and increased amygdala reactivity to negative emotional stimuli compared to those without CT. These findings support the stress sensitization hypothesis, suggesting that vulnerability following CT might be caused by an impaired ability to cope with subsequent stress (McLaughlin et al., 2010). Future research should therefore examine the role of daily stressors in shaping health outcomes. Ecological momentary assessment (EMA) offers a particularly valuable method for capturing real-time stress responses with high ecological validity.

## 9 Conclusions

To summarize, we have shown which (neuro)biological, psychosocial, and behavioral mechanisms may underlie the widespread, transdiagnostic implications of CT, and have raised five key facets imperative to studying and treating (the impact of) CT: I) The interconnectedness of the modalities affected by CT requires increased recognition in research and clinical practice. Considering these systems as a whole provides more comprehensive hypotheses, a more thorough understanding and enhanced treatment options for the adverse impact of CT; II) The impact of CT spans across mental and somatic disorders and III) healthy individuals. Therefore, it should also be considered independent of current health status and diagnostic categories; IV) Precision and consistency in assessing CT, including information on subtypes, duration, timing, and chronicity, are essential to improve the comparability and interpretation of findings; and V) Research should aim to incorporate the impact of (daily life) stress to provide a more comprehensive understanding of the impact of CT. All in all, the multimodal impact of CT requires increased recognition as a starting point for understanding, treating and eventually preventing mental and somatic health problems.

## CRediT authorship contribution statement

**J.M. Pasteuning:** Writing – review & editing, Writing – original draft, Visualization, Methodology, Conceptualization. **C. Broeder:** Writing – review & editing, Writing – original draft, Visualization, Methodology, Conceptualization. **T.A.A. Broeders:** Writing – original draft, Methodology, Conceptualization. **R.G.G. Busby:** Writing – original draft, Methodology, Conceptualization. **A.W. Gathier:** Writing – original draft, Methodology, Conceptualization. **E. Kuzminskaite:** Writing – original draft, Methodology. **F. Linsen:** Writing – original draft, Methodology, Conceptualization. **C.P. Souama:** Writing – original draft, Methodology, Conceptualization. **J.E. Verhoeven:** Writing – original draft, Methodology, Conceptualization. **M.S.C. Sep:** Writing – review & editing, Writing – original draft, Supervision, Methodology, Conceptualization. **C.H. Vinkers:** Writing – review & editing, Writing – original draft, Supervision, Methodology, Funding acquisition, Conceptualization.

## Funding

This work was supported by ZonMW (grant number 09150171910042).

## Declaration of competing interest

The authors declare the following financial interests/personal relationships which may be considered as potential competing interests: C.H. Vinkers reports financial support was provided by Dutch Research Council. If there are other authors, they declare that they have no known competing financial interests or personal relationships that could have appeared to influence the work reported in this paper.
